# Supplementary material for: Decreased Tissue Omega-6/Omega-3 Fatty Acid Ratio Prevents Chemotherapy-Induced Gastrointestinal Toxicity Associated with Alterations of Gut Microbiome
Source: Int J Mol Sci. 2022 May 10;23(10):5332. doi: 10.3390/ijms23105332 (PMC9140600; doi:10.3390/ijms23105332)
Supplement: Supplementary file 1 [file ijms-23-05332-s001.zip › ijms-1679463-supplementary.pdf]

Supplementary Figures:

Decreased Tissue Omega-6/Omega-3 Fatty Acid Ratio Prevents Chemotherapy-Induced Gastrointestinal Toxicity by Modulating Gut Microbiome

Kanakaraju Kaliannan, Shane O Donnell, Kiera Murphy, Catherine Stanton, Chao Kang, Bin Wang, Xiang-Yong Li, Atul K. Bhan, Jing X. Kang

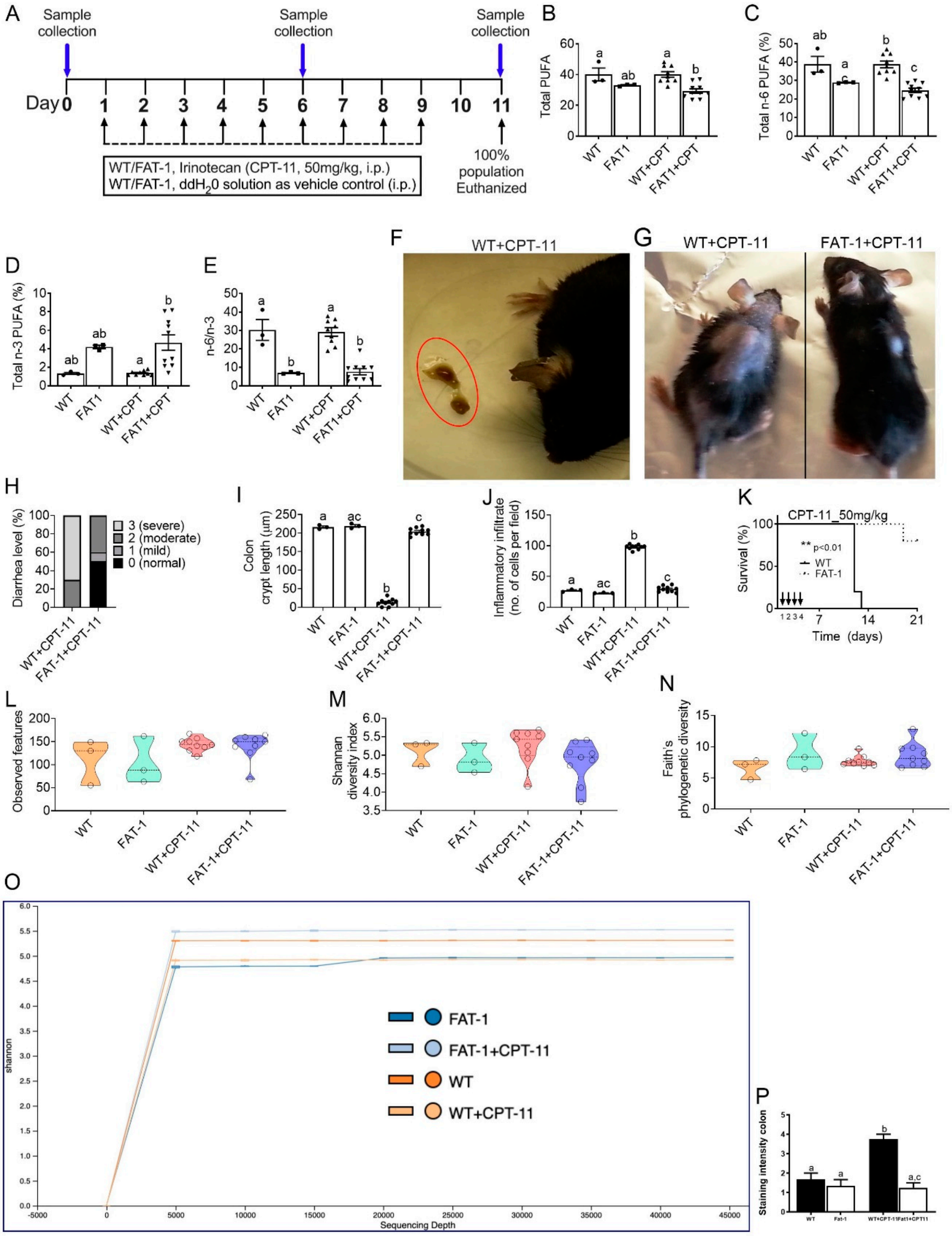

Figure. S1. Decreased tissue n-6/n-3 ratio reduces CPT-11-induced gastrointestinal toxicities and guts microbiome alterations. (*A*) Schematic diagram summarizing the timeline and experimental procedures of this study. WT and FAT-1 transgenic mice were arranged into

four groups, and mice from WT+CPT-11 and FAT-1+CPT-11 groups and WT and FAT-1 control groups were subjected to nine consecutive days of treatment with intraperitoneal injections (i.p) of 50 mg/kg CPT-11 and vehicle (double-distilled water) for CPT-11, respectively. Fecal samples were collected at the baseline before CPT-11 administration and at days 6 after CPT-11 treatment. Cecal contents, serum, and tissue samples were collected on days 11. (***B-E***) Parameters of colon-tissue fatty acids profile such as total polyunsaturated fatty acids (PUFA), total omega-6 (n-6) PUFA, total omega-3 (n-3) PUFA, and n-6/n-3 PUFA ratio. (***F***) Representative picture of bloody diarrhea (red circle) on day 6 in the WT+CPT-11 group. (***G***) Gross appearance of CPT-11 treated WT and FAT-1 mice at the time of euthanasia. (***H***) Differences in diarrhea severity between WT+CPT-11 and FAT-1+CPT-11 groups. (***I***) Crypt length measurements. (***J***) Inflammatory cells infiltrate. (***K***) Differences in the CPT-11–induced lethality in WT and FAT-1 mice treated with CPT-11 (50mg/kg). Kaplan-Meier survival curves and Log-rank (Mantel-Cox) test were used to graph the survival vs. time curves and calculate the P-value. (***L-N***) Violin plots showing the distribution, median (middle line), and quartiles (lines at the top and bottom) values of measures of gut microbiota α-diversity. (***O***) QIIME2 created an alpha rarefaction plot (alpha diversity as a function of sampling depth). (***P***). The staining intensity score was measured from the immunohistochemical analysis of colonic GUSB using the ImageJ software package. Data are shown as mean ± standard error of the mean. Data with different superscript letters are significantly different ($P < 0.05$) according to two-way ANOVA followed by Tukey's multiple comparisons test or Mann Whitney test (**L-N**).

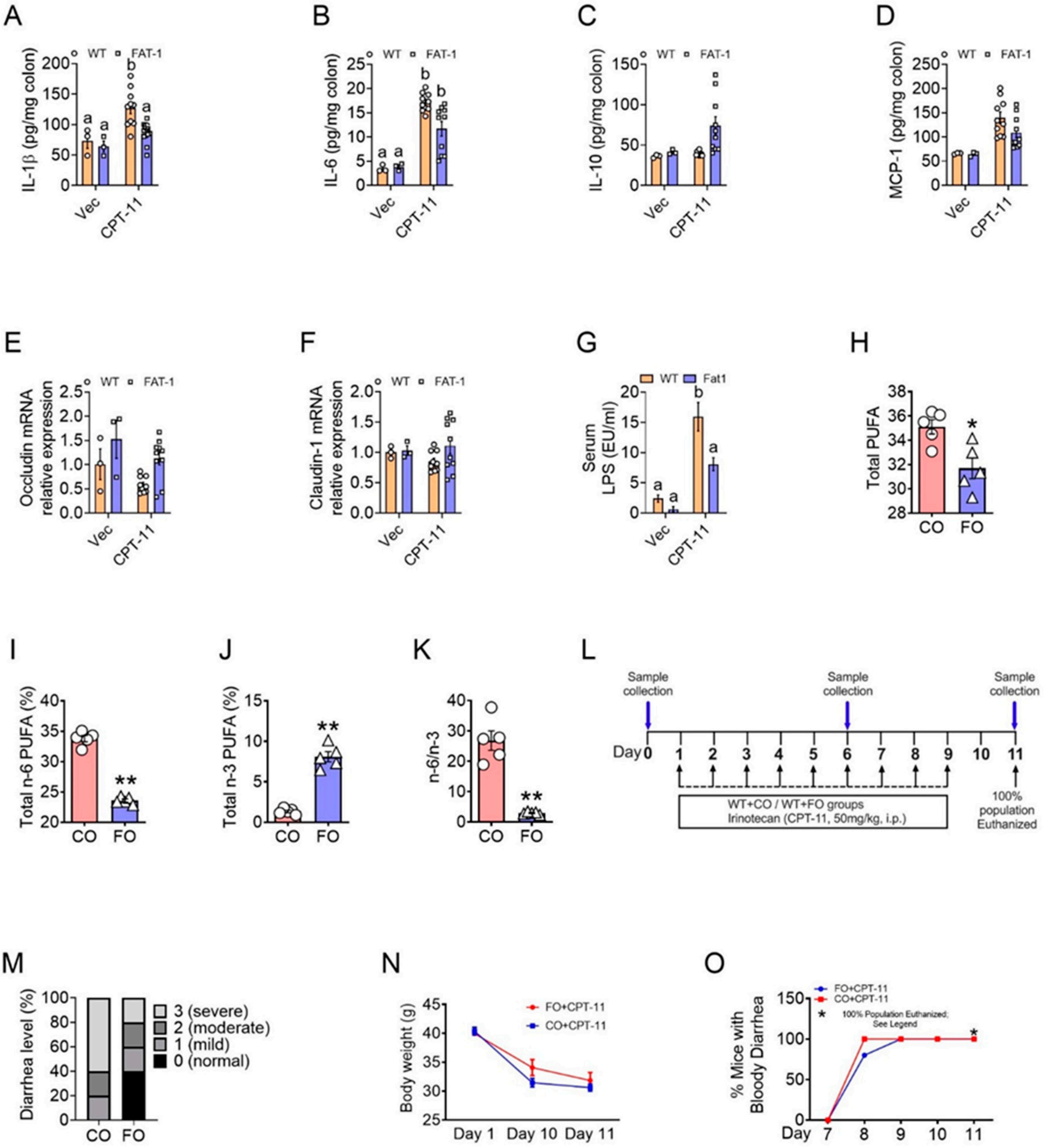

Figure. S2. Decreased n-6/n-3 ratio reduces CPT-11-induced gut-barrier dysfunction. (***A-D***) Colonic tissue levels of Interleukin (IL)-1β (IL-1β), IL-6, IL-10, and monocyte chemoattractant protein-1 (MCP-1). (***E-F***) Differences in the occludin and claudin-1 tight-junction markers relative gene expression (***G***) Serum lipopolysaccharides (LPS) levels (endotoxin units/ml). (***H-K***)

Parameters of colon-tissue fatty acids profile such as total polyunsaturated fatty acids (PUFA), total omega-6 (n-6) PUFA, total omega-3 (n-3) PUFA, and n-6/n-3 PUFA ratio. ***(L)*** Schematic diagram summarizing the timeline and experimental procedures of this study. (***M***) Differences in diarrhea severity between Corn Oil (CO)+CPT-11 and Fish Oil (FO)+CPT-11 groups. A10 days of either corn oil (CO) or fish oil (FO) supplementation to wild-type (WT) male mice followed by CPT-11 (50mg/kg; intraperitoneal route) administration for 11 days. CPT-11-induced gut toxicities such as body weight changes (***N***) and bloody diarrhea (***O***) between CO+CPT-11 and FO+CPT-11 groups. Data are shown as mean ± standard error of the mean. According to one-way ANOVA, data with different superscript letters are significantly different ($P < 0.05$), followed by Tukey's multiple comparisons test or Kruskal-Wallis tests followed by Dunn's multiple comparisons test when the homogeneity of variance was not met with one-way ANOVA. *$P < 0.05$, **$P < 0.01$, ***$P < 0.001$, nonparametric Mann Whitney test. The scale bar for images in C and D panels are 3000 μm.

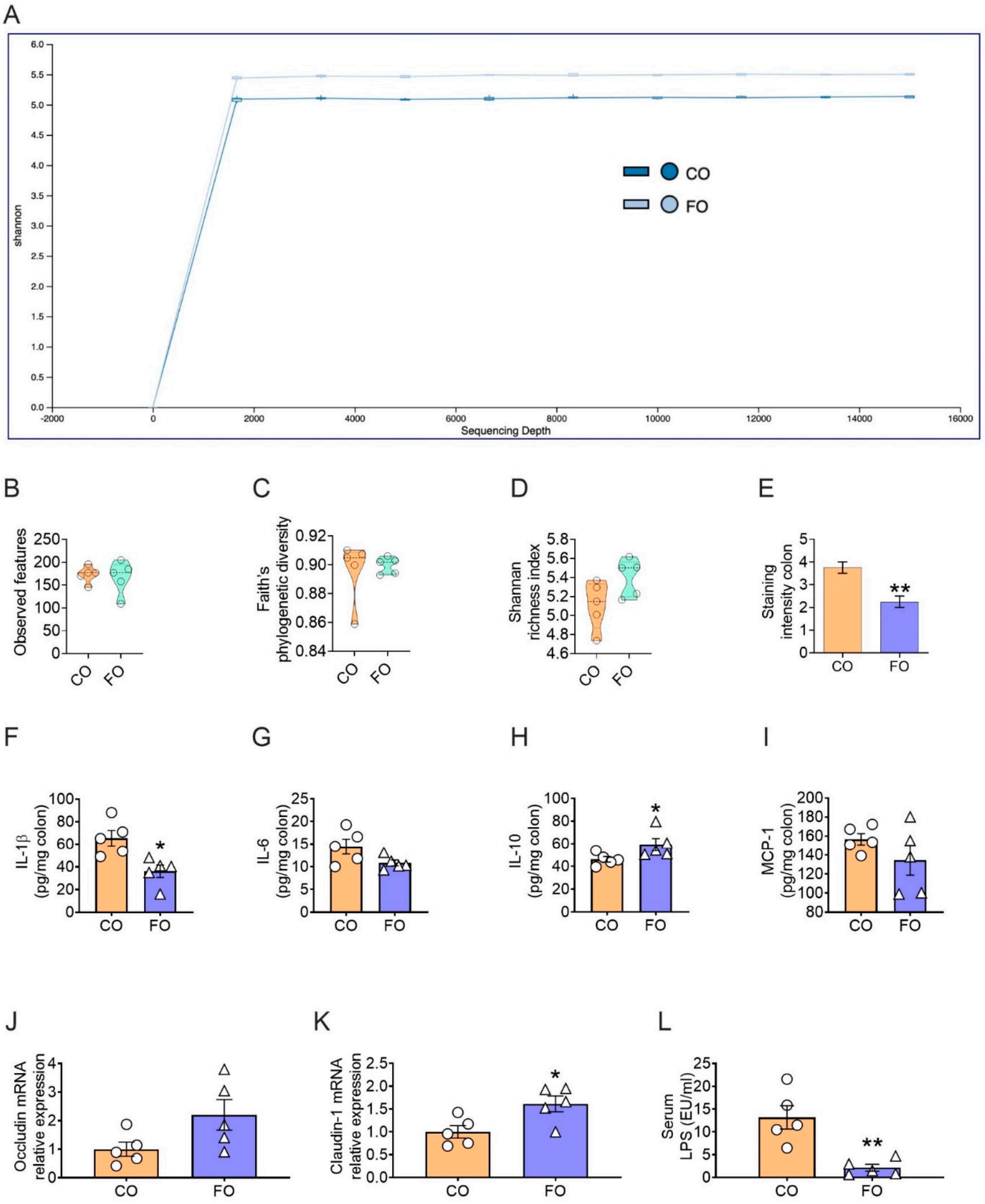

Figure. S3. Dietary n-3 PUFA supplementation reduces CPT-11-induced alterations in the gut microbiome. (***A***) Alpha rarefaction plot (alpha diversity as a function of sampling depth)

was created by QIIME2. (***B-D***) Violin plot with lines at the median (dashed lines) and quartiles (complete lines) showing the differences in the α-diversity measures. (***E***) The staining intensity score was measured from the immunohistochemical analysis of colonic GUSB using the ImageJ software package. (***F-I***) Colonic tissue levels of Interleukin (IL)-1β (IL-1β), IL-6, IL-10, and monocyte chemoattractant protein-1 (MCP-1). (***J-K***) Differences in the occludin and claudin-1 tight-junction markers relative gene expression (***L***) Serum lipopolysaccharides (LPS) levels (endotoxin units/ml). Data are shown as mean ± standard error of the mean. *P < 0.05, **P < 0.01, ***P < 0.001, nonparametric Mann Whitney test.
